# Comparison of the Anti-Obesity Effect of Enriched Capsanthin and Capsaicin from *Capsicum annuum* L. Fruit in Obesity-Induced C57BL/6J Mouse Model

**DOI:** 10.17113/ftb.60.02.22.7376

**Published:** 2022-06

**Authors:** Velmurugan Shanmugham, Ravi Subban

**Affiliations:** Department of Chemistry, Karpagam Academy of Higher Education, Salem-Kochi Highway, Eachanari, Coimbatore, 641 021, India

**Keywords:** capsanthin-enriched pellets, capsaicin pellets, anti-obesity effect, high-fat diet

## Abstract

**Research background:**

Obesity increases mortality and morbidity due to its impact on type 2 diabetes, cardiovascular and gastrointestinal diseases, arthritis and certain cancers. The epidemic of excessive mass and obesity require constant research to improve therapies without undesirable side effects. Therefore, exploring the anti-obesity phytochemicals from food sources is essential. Most pharmacological studies of the anti-obesity potential of *Capsicum annuum* have been directed towards capsaicin and very few towards capsanthin. However, these studies utilized uncoated capsaicin and capsanthin. This study aims to compare the anti-obesity effects of enteric-coated capsaicin and capsanthin in a high-fat diet-induced obesity in animal model.

**Experimental approach:**

In this study, we investigated the anti-obesity properties of capsanthin-enriched pellets and capsaicin pellets derived from red chili fruit (*Capsicum annuum*) on high-fat diet (HFD)-induced obesity in C57BL/6J mice. First, the animals received HFD to induce their obesity. Animals were supplemented orally with pellets. The food intake, body mass, obesity and clinical biomarkers were assessed.

**Results and conclusions:**

The mice fed with HFD gained body mass and white adipose tissue mass compared to the mice that consumed a normal diet. The oral administration of capsanthin-enriched pellets and capsaicin pellets significantly reduced the body mass gain. These pellets have a statistically significant (p<0.05) impact on obesity biomarkers by increasing adiponectin and decreasing leptin, free fatty acid and insulin concentrations relative to HFD control. There was no change in the liver mass in all groups, but there was a significant decrease in white adipose tissue amounts. Inguinal adipose tissue amount was reduced by 37.0% and that of epididymal adipose tissue by 43.64% after treatment with capsanthin-enriched pellets. These results suggest that capsanthin-enriched pellets and capsaicin pellets may be useful in combating metabolic diseases, including obesity, without adverse effects.

**Novelty and scientific contribution:**

We increased the content of capsanthin for more than 50% in capsanthin-enriched extract and extended the room temperature stability for more than one year by converting the crystals into capsanthin-enriched pellets. This study breaks new ground by examining the potential of capsanthin >50% in the management of obesity for the first time.

## INTRODUCTION

Obesity alone is associated with an increased risk of morbidity and mortality (*1*), but with the COVID-19 pandemic, the condition has more severe consequences. Obesity is an important risk factor associated with mortality in people infected by COVID-19 (*2*) due to the possible long-term, persistent and irreversible undesirable effects of the virus on obesity (*3*). With the new pandemic, obesity might impact most cardiovascular disease (CVD) risk factors, including hypertension, *diabetes mellitus* and abnormalities in blood glucose with undesirable effects on the cardiovascular system (*4*). Chronic inflammatory pulmonary disease and bronchial asthma are additional morbidities associated with obesity (*5*). The potential of having type 2 diabetes and almost all forms of CVD, especially heart failure, hypertension, coronary heart disease, atrial fibrillation and peripheral arterial diseases (*6*), is increased due to the rise in central adiposity with visceral adipose tissue (*7*). Adipose tissues are responsible for energy storage and function as an endocrine organ known to secrete adipokines. The pro- and anti-inflammatory activities of adipokines are linked to obesity-related complications (*8*).

In recent years, the demand for dietary antioxidants, especially phytochemicals, has tremendously increased owing to their beneficial role in adiposity (*9*). Although various kinds of anti-obesity medications are available, these are associated with severe side effects (*10*). Therefore, exploring new phytochemicals with the potential to combat obesity without undesirable side effects is vital. The beneficial role of carotenoids in the management of obesity has been studied using a natural carotenoid mixture and vitamins (*11*). The most studied carotenoids in lipid metabolism are β-carotene, cryptoxanthin, astaxanthin and fucoxanthin; moreover, recently, capsanthin has been gaining more attention. In the prevention of adiposity and obesity, the role of carotenoids and carotenoid derivatives is promising (*12*). Carotenoids are highly unstable, especially at higher purity. Their degradation is followed by oxidation due to the characteristic conjugated polyene chain present in its structure. Sometimes, the terminal groups also suffer degradation under certain environmental conditions. They also require storage under nitrogen and refrigerated conditions for their assured shelf life.

Therefore, the engineering of carotenoids to ensure stability is crucial (*13*). In order to maintain stability throughout their shelf life, encapsulation by various polymers as coating agents has emerged as an excellent technique (*14*).

Red bell pepper (*Capsicum annuum* L.) has a long history of use as a vegetable, food colour, hot flavoring agent and in traditional medicines (*15*). The effect of red paprika capsanthin on obesity in both animal and human models has been reported, but no study has used highly enriched capsanthin. For the first time, we have obtained above 50% capsanthin in capsanthin-enriched extract and extended the room temperature stability of capsanthin-enriched pellets for more than one year. Moreover, we evaluated the anti-obesity effect of capsanthin-enriched pellets and capsaicin pellets derived from red bell pepper fruit in a high-fat diet (HFD) induced obesity model using male C57BL6/J mice. The anti-obesity effects of the oral administration of capsanthin-enriched pellets in three dosage groups per body mass: low, medium and high (20.5, 40.1 and 82 mg/kg respectively) were compared to those of capsaicin pellets in one dose group of 16 mg/kg and control groups (normal and high-fat diets without any additions).

## MATERIALS AND METHODS

### Chemicals

Enteric-coated capsaicin (2%) pellets were provided by Phytosol India Pvt Ltd, Bangalore, India. Sodium alginate (Vivapharm®; JRS PHARMA GmbH & Co. KG, Rosenberg, Germany), microcrystalline cellulose powder (Pharmacel® 101; DFE Pharma, Goch, Germany) and ethyl cellulose (ASHACEL® M-20; Asha Cellulose Pvt Ltd, Abrama, Valsad, Gujarat, India) were used. Besides, *n*-hexane, ethyl acetate, isopropyl alcohol, polyvinyl pyrrolidine K-30, sucrose and sodium dodecyl sulfate were procured from Sigma-Aldrich, Merck, Bangalore, India. Orlistat 97% purity was purchased from TCI Chemicals Pvt. Ltd, Chennai, India. Normal fat diet and high-fat diet from Research Diet Inc, New Brunswick, NJ, USA were used in this study. The reference standard capsanthin was purchased from Sigma-Aldrich, Merck, St. Louis, MO, USA.

### Capsanthin-enriched extract

Red bell pepper (*Capsicum annuum*) fruits were procured from the local market in Bangalore, India, dried, deseeded and flaked. The flaked fruits were extracted with *n*-hexane (*16*), then filtered and concentrated to obtain the oleoresin. The oleoresin was further purified by supercritical extraction and subjected to alkaline saponification. The saponified extract was washed with water and purified with ethyl acetate to obtain capsanthin-enriched extract (*17*).

### Capsanthin-enriched pellets

In a glass bowl, capsanthin-enriched extract, sucrose and microcrystalline cellulose were mixed geometrically, and 5% (*m/V*) polyvinyl pyrrolidine K-30 solution in isopropyl alcohol was added to form a coherent mass. Wet pellets from the coherent mass were prepared by using a twin-screw axial extruder (Anish Pharma Equip Pvt. Ltd., Nashik, India). The wet pellets were transferred to a spheronizer (*18*) and dried. Pellets were coated with sodium alginate and ethyl cellulose barrier using a fluidized bed coater with bottom spray (Anish Pharma Equip Pvt. Ltd.). The pellets were removed and cured at room temperature.

### Physical, chemical and microbial analyses of *capsanthin-enriched pellets*

The physical, chemical and microbial analyses were performed using the methods described in the United States Pharmacopeia (USP). The micrographs of capsanthin-enriched extract and pellets, and capsaicin pellets were captured by Canon digital camera (SX160 IS; Cannon USA Inc., Melville, NY, USA). The solubility of the capsanthin-enriched pellets (1 g) in alcohol and water was analyzed using USP method 561 (*19*). The particle size was determined by dry sieve analysis as per the method described in USP 786 (*20*). In this method, 5 g of pellets passed through US sieve number 30. The loss on drying was determined by drying 1 g of pellets at 105 °C to constant mass as per USP 731 (*21*) procedure.

The Joint Ethical Committee on Food Additives (JECFA) method was employed to quantify capsanthin and carotenoids (*22*). As per the method, capsanthin was extracted using acetone, then saponified and subjected to high-performance liquid chromatography (HPLC). The HPLC chromatograph equipped with LC solution software (model LC2030C, i-Series plus; Shimadzu, Kyoto, Japan) was used. The C18 reversed-phase column with the dimensions 250 mm×4.6 mm, 5 microns was used. The binary gradient mobile phase consisted of acetone (A) and water (B). The gradient program was set at 0 to 5 min 75% B, 5 to 10 min 75–95% B, 10 to 17 min 95% B, 17 to 22 min, 95-100% B, 22 to 75 min 100-75% B. The total flow rate was maintained at 1.2 mL/min, and peaks were detected at 474 nm. The standard and the samples were prepared in acetone and sonicated for 10 min. The retention time of capsanthin in the standard was used to identify the capsanthin in capsanthin-enriched extract and pellets. Carotenoids were estimated by spectrophotometry. The UV spectrophotometer equipped with Lab solution software (model 1900i; Shimadzu) was used. A suitable quantity of pellets was dissolved in acetone and the absorbance was measured at 462 nm (*22*).

The elemental impurities like lead, arsenic, cadmium and mercury were estimated by using USP 233 (*23*) method. In this method, inductively coupled plasma-optical emission spectroscopy (ICP-OES) equipment (model 5510; Agilent Technologies Inc., Santa Clara, CA, USA) was used. The ICP-OES instrument conditions were argon plasma 15 L/min, auxiliary gas 0.9–1.0 L/min, analysis mode 500-700 kPa and nebulizer gas maintained at 0.7–1.2 L/min.

The microbiological parameters of the pellets were also assessed by USP procedures. The total plate, yeast and mould counts were determined by USP 2021 method (*24*) using 1 g pellets. Pathogens such as *Escherichia coli*, *Salmonella* sp. and *Staphylococcus aureus* were analyzed as per method USP 2022 (*25*) using 10 g pellets. The *Pseudomonas aeruginosa* analysis was performed as per USP method 62 (*26*) with 10 g sample.

### Dissolution study

The *in vitro* release profile of capsanthin-enriched extract and pellets was quantified using dissolution apparatus (model DS 8000; Lab India Analytical Instruments, Bangalore, India) and following the dissolution method described in USP 711 method (*27*). The experiment adopted the paddle method, with the addition of 900 mL of phosphate buffer at pH=6.8 and 1% sodium lauryl sulfate as the medium, at 100 rpm rotating speed and 37 °C, to determine the dissolution of capsanthin. About 5.0 mL of each sample were withdrawn at regular time intervals and replaced by the fresh medium. Capsanthin was quantified by the HPLC method described above.

### Stability study

The stability study was conducted as per ICH guidelines Q1A (R2) (*28*). Optimized formulations of capsanthin-enriched pellets were placed in a low-density polyethylene bag, packed in high-density polyethylene (HDPE) containers. The containers were kept in a stability chamber, and temperature ((25±2) °C) and relative humidity ((60±5) %) were maintained. The samples were analysed initially and after the end of a period of 3, 6, 9 and 12 months (*28*). The following parameters were examined: change in the appearance, *i.e.* colour, identification and content of capsanthin and carotenoids by HPLC.

### Isolation of pure capsanthin by preparative HPLC

A spectroscopically pure capsanthin was isolated from partially purified capsanthin-enriched extract. The separation was performed with a preparative HPLC (model 1200; Agilent Technologies Inc.). The pure capsanthin fraction was characterized by LC-MS, ^1^H-NMR and ^13^C-NMR.

### LC-MS analysis

The preparative HPLC fraction 3 (F3) was analyzed to determine the mass by in-house developed LC-MS with a binary gradient elution using Waters LCMS Micromass ZQ quadrupole mass analyzer coupled with tandem HPLC (model e2695, Xevo G2-Xs QTof; Waters Corporation, Milford, MA, USA).

### ^1^H and ^13^C NMR characterization

^1^H and ^13^C nuclear magnetic resonance spectra (*29*) were recorded to determine the structure of the isolated capsanthin on Agilent-NMR (model VNMRS-400; Agilent Technologies Inc.). The samples were prepared in deuterated chloroform. Various proton environments were monitored by analysing an H-H correlation within the chemical structure.

### Animals and experimental protocol

The Institutional Animal Ethics Committee (IAEC) approved this *in vivo* study protocol with approval number VIP/IAEC/156/2019. In this study we implemented the measures recommended by the CPCSEA (Committee for Control and Supervision of Experiments on Animals) (*30*). The four-week-old male C57BL/6 mice (Hylasco, Hyderabad, India) were acclimatized for one week to a specific temperature ((22±3) °C), humidity ((30-70)±5) %) and lighting 08:00–20:00 conditions after their procurement. Polycarbonate cases were used to house the animals, and free access to drinking water and food was provided. The C57BL/6 mice were randomly divided into seven groups of ten mice each after adaptation. Group G1 (normal diet, ND) was fed with a 42% kJ fat diet, and the remaining groups (G2 to G7) were fed with a 251% kJ fat diet to induce obesity for seven weeks. The induction of obesity was confirmed by body mass increase. The capsanthin-enriched pellets were suspended in 0.5% carboxymethyl cellulose (CMC) and administered orally to groups on body mass basis: G3 (20.5 mg/kg, *i.e.* low dose), G4 (41 mg/kg, *i.e.* medium dose) and G5 (82 mg/kg, *i.e.* high dose) for five weeks. Groups G6 and G7 received capsaicin pellets (16 mg/kg) and orlistat (16 mg/kg) in 0.5% CMC, respectively, for five weeks. The normal diet control (G1) and high-fat diet (HFD) control (G2) mice were provided with only 0.5% CMC. The body mass and food intake were measured twice weekly during the obesity induction period and thrice weekly thereafter up to the end of the treatment.

### Clinical pathology

At the end of the study, blood samples were collected for haematology, clinical pathology investigations and biomarker analysis. The haematology analyzer (model ABX Micros ESV 60; HORIBA Instruments Inc., Irvine, CA, USA) was used for haematological evaluation. Clinical chemistry parameters were estimated using the RX Daytona+ (Randox Laboratories, Kearneysville, WV, USA) instrument. Commercially available enzyme-linked immunosorbent assay kits (KinesisDx kits; Krishgen Biosystems, Mumbai, India) were used to quantify insulin, leptin, adiponectin and free fatty acids. At the end of the study, all animals were subjected to a detailed gross necropsy examination. External body surfaces, orifices and cavities such as abdominal, thoracic and cranial were included in the examination.

### Histopathology and necropsy

When the body mass was measured, the liver, abdominal adipose tissues (inguinal and white epididymal) and kidney tissues were fixed in 10% natural buffered formalin, stained with hematoxylin and eosin (Everon Life Sciences, New Delhi, India), and subjected to histopathological evaluation. Adipose tissues were observed microscopically (at 40×, BX53; Olympus Corporation, Shinjuku-ku, Japan) for any change in adipocytes. Both internal and external gross necropsy examinations were done on all the tested animals.

### Statistical analysis

A one-way analysis of variance (ANOVA) was used for multiple comparisons. The effects of treatment compared to control were analyzed using the GraphPad software, v. 8.0 (*31*). The values were expressed as mean±S.D. (standard deviation), compared and evaluated at a 5% (p≤0.05) level.

## RESULTS AND DISCUSSION

**Fig. S1:**
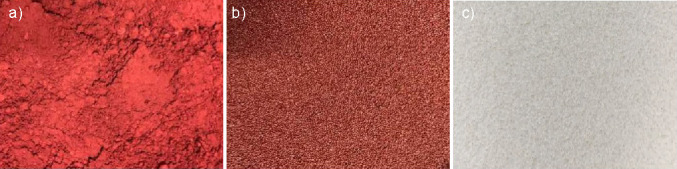
The appearance of: a) capsanthin-enriched extract, b) capsanthin-enriched pellets, and c) capsaicin pellets. 10× magnification

In our study, capsanthin was enriched to above 50%, which extended the room temperature storage stability for more than one year by converting it into capsanthin-enriched pellets. Fig. S1 shows the appearance of capsanthin-enriched extract, capsanthin-enriched pellets and capsaicin pellets. The used pellet and coating compositions are shown in Table S1, and the specification for capsanthin-enriched pellets is shown in Table S2. The capsanthin-enriched pellets comply with all the parameters mentioned in Table S2.

### In vitro drug release

**Fig. S2:**
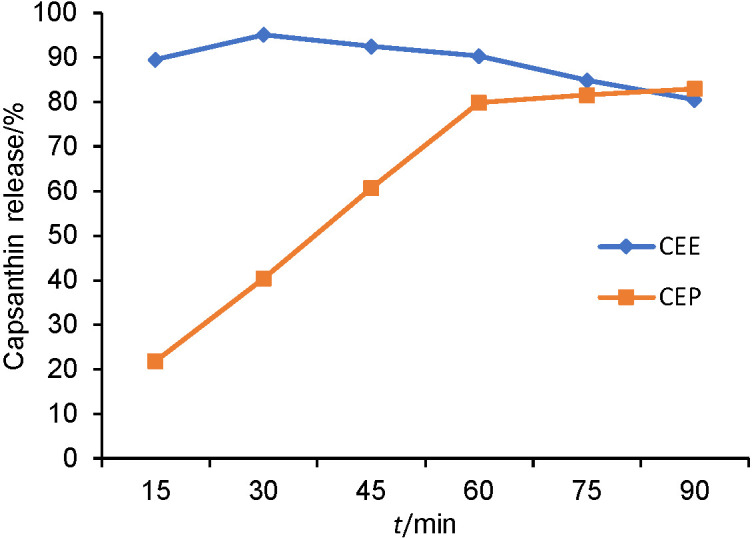
*In vitro* drug release of capsanthin-enriched extract and capsanthin-enriched pellets. CEE=capsanthin-enriched extract, CEP=capsanthin-enriched pellets

The hydrophilic matrix formulation of capsanthin-enriched pellets was developed using microcrystalline cellulose, sucrose and polyvinyl pyrrolidine K 30 and further coated with sodium alginate and ethyl cellulose as barrier. The polymers on the exterior surface hydrate and swell, forming a protective gel layer and slowly and steadily release capsanthin. The release of capsanthin is due to either diffusion or erosion of the gel layer or the combination of both processes (*32*). The *in vitro* cumulative capsanthin release profiles of capsanthin-enriched extract and capsanthin-enriched pellets using the dissolution apparatus in the buffer at pH=6.8 are shown in Fig. S2. As can be seen, the capsanthin-enriched extract and capsanthin-enriched pellets exhibited different release patterns. The release profile of capsanthin-enriched extract was observed to be rapid, while that of capsanthin-enriched pellets occurred in a sustained release manner.

### Stability of capsanthin-enriched pellets

The physical and chemical stability of capsanthin-enriched pellets was evaluated as per the ICH guidelines (*28*). At the end of one year, no change in the physical characteristics such as degradation or change in the colour was observed. Chemical analysis revealed that no degradation in the content of total carotenoids and capsanthin was observed. In conclusion, capsanthin-enriched pellets were stable at 25 ºC and 60 % relative humidity. The stability data are presented inTable S3.

### Spectral characterization

**Fig. S3:**
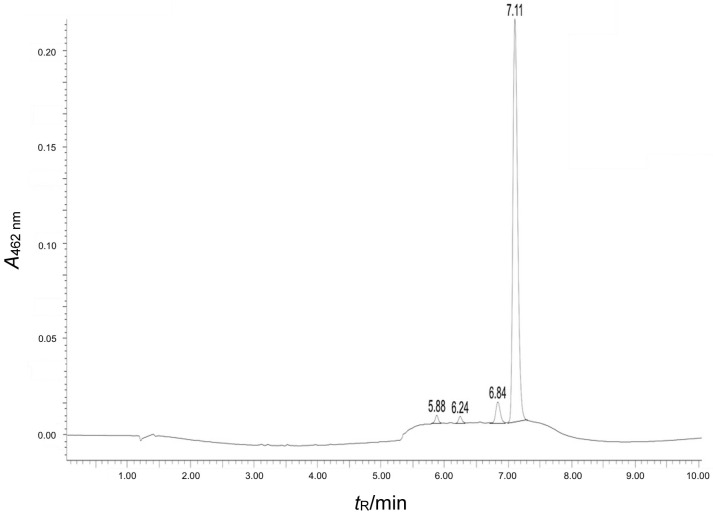
HPLC chromatogram of capsanthin-enriched extract. Retention time 7.11 min corresponds to capsanthin

**Fig. S4:**
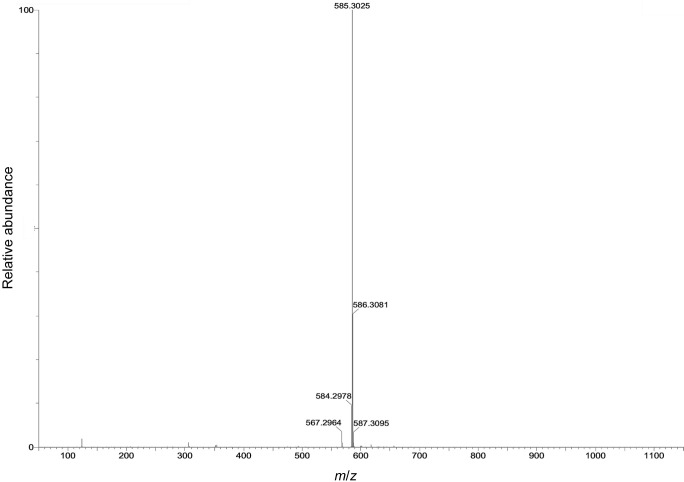
Mass spectrum fragmentation of capsanthin (*t*_R_=7.122 min)

**Fig. S5:**
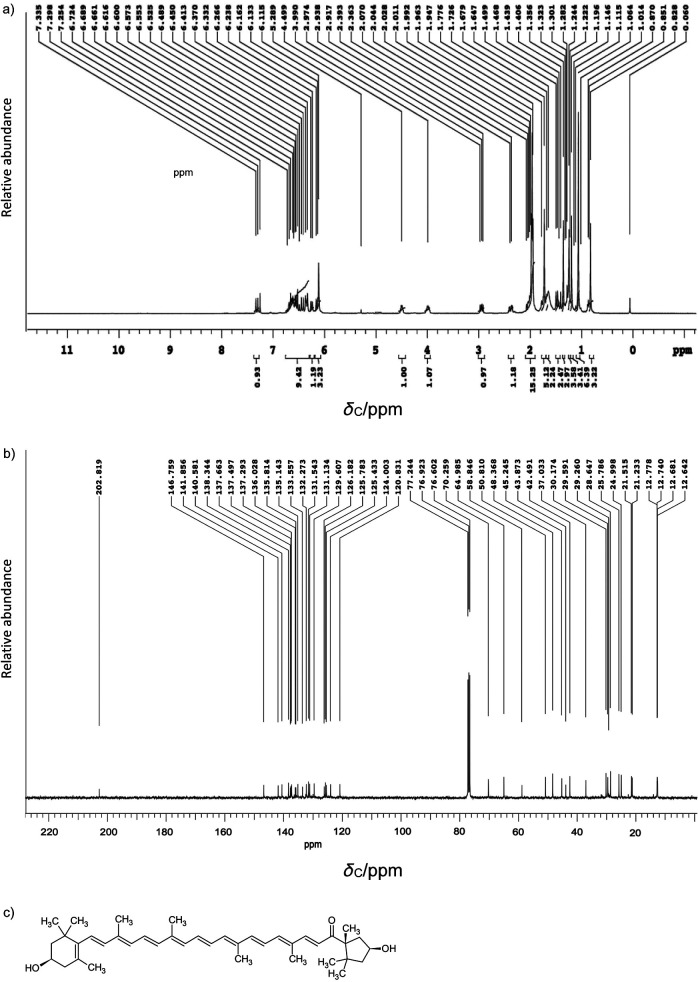
Capsanthin characterization: a) ^1^H NMR spectra, b) ^13^C NMR spectra, and c) structure

LC-MS/MS with HPLC technique was employed to determine the chemical profile of capsanthin-enriched extract. Liquid chromatogram patterns of capsanthin-enriched extract showed major peaks at their retention times of 5.88, 6.24, 6.84 and 7.11 min (Fig. S3). Furthermore, capsanthin-enriched extract showed a molecular ion peak at a retention time of 7.11 min with the fragmentation of *m*/*z*=585.30 [M^+^]^+^ (Fig. S4) and dehydrated productions ([MH-H_2_O]^+^ at *m*/*z*=567, which was correlated with capsanthin from literature studies (*33*). In order to unequivocally prove the capsanthin identification, after isolation from capsanthin-enriched extract, the structure was confirmed using ^1^H-NMR and ^13^C-NMR spectroscopy. The NMR data were consistent with those in the literature (*33*). The ^1^H-NMR, ^13^C-NMR data and the structure of capsanthin are shown in Figs. S5a, S5b and S5c respectively.

### The anti-obesity effect of capsanthin-enriched pellets and capsaicin pellets on HFD-induced diabetes in C57BL/6J mice

#### Effect of capsanthin-enriched pellets on body mass

The effect of capsanthin from red bell pepper on adipogenesis and mass gain inhibition in HFD-induced diabetes in animal models was reported by Kim *et al.* (*34*), who used a low mass per volume ratio of 0.005% capsanthin, and Jo e*t al.* (*35*), who used a mixture of transesterified carotenoids with a maximum amount of 10 μmol capsanthin. In this study, the anti-obesity activity of capsanthin-enriched pellets (>50% capsanthin) on HFD-induced obesity in C57BL/6 mice was evaluated and compared to HFD control, capsaicin pellets 2% and the reference drug orlistat.

**Fig. 1:**
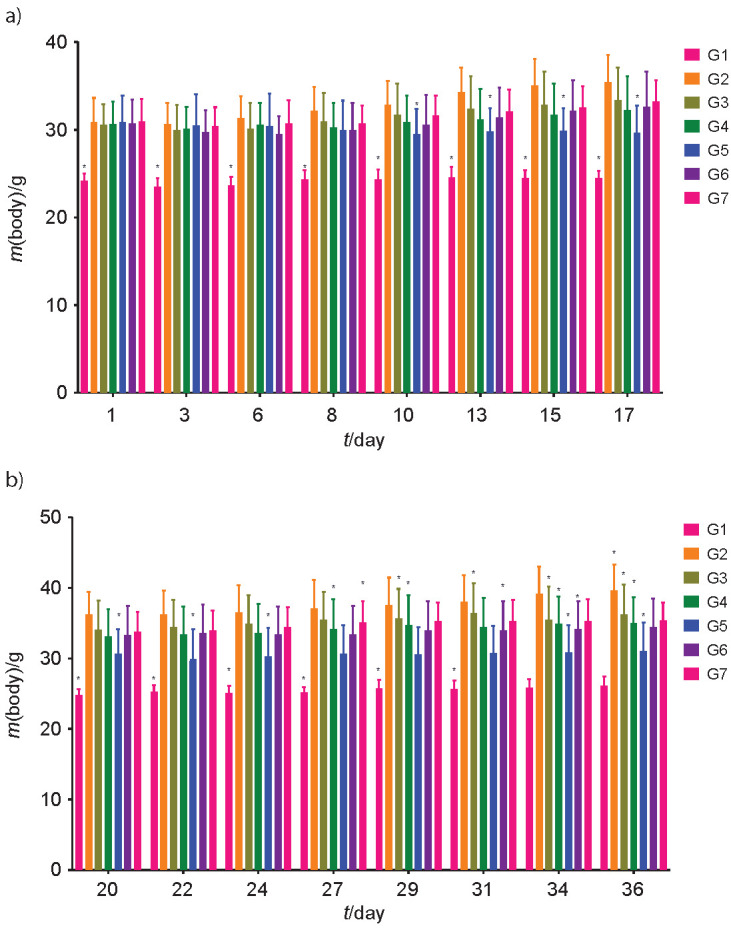
Comparison of body mass in control and treated groups: a) days 1 to 7, and b) 20 to 36. G1=normal control, G2=HFD control, G3=HFD+capsanthin-enriched pellets (low dose), G4=HFD+capsanthin-enriched pellets (medium dose), G5=HFD+capsanthin-enriched extract pellets (high dose), G6=HFD+capsaicin pellets, G7=HFD+orlistat, HFD=high fat diet. Values are expressed as mean±standard deviation. *Indicates statistically significant differences (p<0.05) with respect to G2, *N*=10

HFD-induced obesity was confirmed after the mice were fed with HFD for seven weeks. The HFD group showed a statistically significant body gain (p<0.05), and the mean body mass gain was 164.1% compared to the normal diet (ND) group (Fig. 1). A statistically significant (p<0.05) mass gain reduction was observed in the groups fed HFD in combination with capsanthin-enriched pellets and HFD in combination with capsaicin pellets compared to the HFD control. The mass gain reduction in the groups fed low, medium and high dose of HFD with capsanthin-enriched pellets was 34.70, 51.14 and 98.51%, respectively. These values can be compared to the groups fed HFD with capsaicin pellets (57.15% lower) and HFD with orlistat (48.86% lower). Our study validated the mass gain inhibition action of capsanthin in an animal model as previously reported by Kim *et al.* (*34*) and Jo *et al.* (*35*) but could not compare the efficacy of our capsanthin to previously reported studies due to a lack of sufficient information and a statistically not significant mass gain reduction reported by Jo *et al.* (*35*). Wu *et al.* (*36*) reported the mass gain reduction of 27.5% in the HFD-induced obese model, but the study involved low mass fractions of pure capsanthin extract. A significantly higher food intake was observed in the ND group than in the HFD group, but there was not much variation between the control and the treated groups, as shown in Figs. 1a and 1b respectively. The summary of food intake is shown in Table S4.

#### Effect of capsanthin-enriched pellets on HFD-induced changes in adiponectin concentrations in mice

**Fig. 2:**
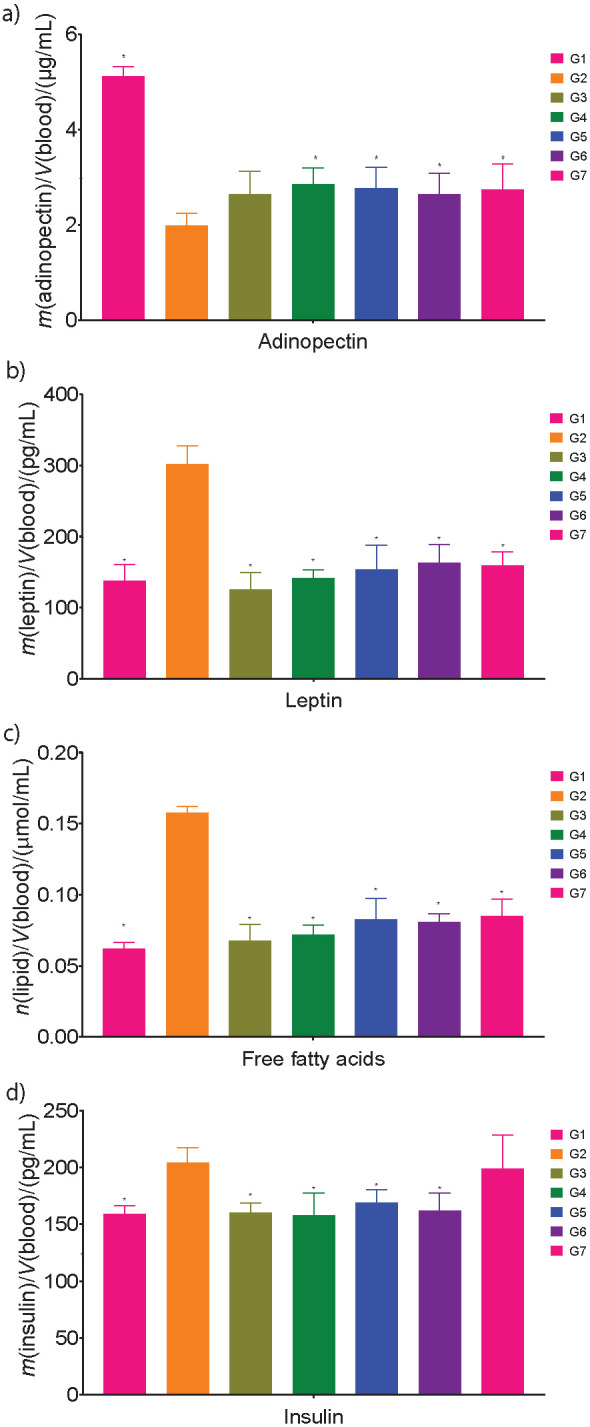
Effect of capsanthin on obesity biomarkers: a) insulin, b) leptin, c) adiponectin, and d) free fatty acids. G1=normal control, G2=HFD control, G3=HFD+capsanthin-enriched pellets (low dose), G4=HFD+capsanthin-enriched pellets (medium dose), G5=HFD+capsanthin-enriched pellets (high dose), G6=HFD+capsaicin pellets, G7= HFD+orlistat. HFD=high fat diet. Values are expressed as mean±standard deviation. *Indicates statistically significant differences (p<0.05) with respect to G2, *N*=10

Adiponectin is one of the adipokines, predominantly secreted by adipose tissues in adult humans, with insulin-sensitizing action through adiponectin receptors (Adipo R1 and Adipo R2) and anti-inflammatory and anti-apoptotic properties (*37*). Adiponectin enhances insulin sensitivity and signalling by activating *AMPK, PPAR-γ* and other unknown pathways (*38*). In skeletal muscles, adiponectin increases *CD36*, and acyl-coenzyme-A oxidase expression leads to a reduction in the free fatty acid and triglyceride levels (*39*). Furthermore, adiponectin ensures an increase in glucose uptake, and fatty acid combustion leads to a reduction in blood glucose (*40*). In all groups fed HFD with capsanthin-enriched pellets, the adiponectin concentration increased significantly (p<0.05). The adiponectin concentrations in groups fed HFD with low, medium and high doses of capsanthin-enriched pellets were (2.65±0.47), (2.86±0.34) and (2.77±0.44) µg/mL respectively, and in the group fed HFD, the adiponectin level was (1.98±0.26) µg/mL (Fig. 2a). A similar trend was observed in the groups fed HFD with capsaicin pellets ((2.64±0.44) µg/mL) and HFD with orlistat ((2.74±0.74) µg/mL). Overall, in the group fed a high dose of HFD with capsanthin-enriched pellets, a significant increase in the adiponectin content (28.02% higher) was observed compared to the HFD control group and this is remarkable when compared to Kim *et al.* (8.65% increase) (*34*) and Jo *et al.* (statistically not significant increase) (*35*).

#### Effect of capsanthin-enriched pellets on HFD-induced changes in leptin concentrations in mice

Leptin, another adipokine that regulates energy expenditure and food intake, is encoded by the obese gene and is closely related to the adipose tissue mass and body mass index (BMI) in humans (*41*). In rodents and humans, a linear correlation was noted between leptin concentrations and mass loss. Elevated leptin concentration in plasma is the leading cause of obesity in humans. The plasma leptin concentration may be an indicator of body fat increase (*42*). Capsanthin reduces serum leptin concentration in HFD-induced obesity in animal model as reported by Kim *et al*. (*34*), which is confirmed by our study. The high-fat diet significantly increased (p<0.05) the blood plasma leptin concentration compared to control group (Fig. 2b). The leptin concentrations in groups fed HFD and ND were (301.99±25.60) and (137.98±22.68) pg/mL respectively. A statistically significant (p<0.05) decrease of leptin was observed in all the groups fed HFD with capsanthin-enriched pellets compared to HFD control. The leptin concentration in groups treated with low, medium and high doses of capsanthin-enriched pellets were (125.21±24.59), (141.39±12.13) and (153.92±33.68) pg/mL respectively. In the same manner, the groups fed HFD with capsaicin pellets and HFD with orlistat showed a statistically significant decrease in leptin concentrations compared to the HFD control group. The leptin concentrations in the groups fed HFD with capsaicin pellets and HFD with orlistat were (162.62±26.15) and (159.67±19.13) pg/mL, respectively. In the groups fed HFD with capsanthin-enriched pellets, there was a statistically significant (p<0.05) decrease in leptin (49.03% lower) concentration compared to the HFD control group and 19.54% reduction of leptin reported by Kim *et al.* (*34*). The same trend was noted in the groups fed HFD with capsaicin pellets (46.15% lower) and HFD with orlistat group (47.13% lower).

#### Effect of capsanthin-enriched pellets on HFD-induced changes in free fatty acid concentrations in mice

Scientific studies have established the relationship between elevated plasma free fatty acids (FFA), also referred to as non-esterified fatty acids (NEFA), and several metabolic disorders such as insulin resistance (*43*). Adipose tissues are responsible for the storage and release of free fatty acids. In obesity, enlarged adipose tissue and reduction in FFA clearance are responsible for elevated plasma FFA. In addition, antilipolytic action of insulin inhibited by FFA causes further release of the FFA into the circulation. Increasing plasma FFA concentration in skeletal muscle reduces insulin-stimulated glucose uptake. In the body, more than 80% of glucose uptake occurs in skeletal muscle. In the liver, the suppression of hepatic glucose production is due to FFA-induced insulin resistance. Chronically, elevated plasma FFA concentrations lead to insulin resistance in obese diabetic and non-diabetic individuals. Studies have demonstrated that normalizing plasma FFA concentrations resulted in insulin-stimulated glucose uptake in obese individuals with diabetes (*44*). In obesity, impaired suppression of hepatic glucose output is caused by insulin resistance and a decrease in glucose transport (*45*). The action of capsanthin on FFA was previously reported by Kim *et al*. (*34*), and our study showed a similar finding.

In this study, groups receiving HFD with capsanthin-enriched pellets showed a statistically significant (p<0.05) decrease in plasma FFA concentrations compared to the HFD control group (Fig. 2c). The HFD groups had higher FFA concentrations ((0.1577±0.0044) µmol/mL) than ND groups ((0.0621±0.0046) µmol/mL). The FFA concentrations in groups fed low, mid and high doses of HFD with capsanthin-enriched pellets were (0.0677±0.0113), (0.0717±0.007) and (10.0827±0.0149) µmol/mL respectively, and these values are close to those of the ND groups. The FFA concentration was (0.0807±0.0060) µmol/mL in the group receiving HFD with capsaicin and (0.0851±0.0120) µmol/mL in the group receiving HFD with orlistat, and these values are comparable to those of the groups receiving HFD with capsanthin-enriched pellets. A statistically significant (p<0.05) reduction in free fatty acids was observed in the group receiving a high dose of HFD with capsanthin-enriched pellets and the reduction was 47.55% compared to HFD. In our study, the reduction in FFA is quite significant compared to the study reported by Kim *et al*. (*34*), where the reduction was 12.76%.

#### Effect of capsanthin-enriched pellets on HFD-induced changes in insulin concentrations in mice

Kim *et al*. (*34*) showed that the supplementation of capsanthin significantly reduced insulin concentrations compared to the group without treatment. Similarly, in our study, groups treated with capsanthin-enriched pellets showed a statistically significant (p<0.05) reduction in insulin concentration compared to the HFD control group (Fig. 2d). The insulin concentration of the control groups fed ND or HFD was (158.77±7.53) and (204.20±13.12) pg/mL respectively. The insulin concentration in the groups receiving low, medium and high doses of HFD with capsanthin-enriched pellets was (160.02±8.90), (157.81±19.39) and (168.79±11.77) pg/mL respectively. The group fed HFD with capsaicin pellets had the insulin concentration of (162.19±15.36) pg/mL, which is statistically significant compared to the HFD control. A nonsignificant change in the insulin concentration of (198.70±29.56) pg/mL was observed in the group fed HFD with orlistat and was almost equal to that of the HFD control. A statistically significant (p<0.05) reduction in insulin was noted in the group treated with a high dose of HFD with capsanthin-enriched pellets, which was 49.03% compared to HFD control group. Insulin reduction reported by Kim *et al.* (*34*) was 9.99%.

### Gross necropsy and histopathology

All surviving animals were subjected to necropsy and both external and internal gross pathological examinations at the end of the study. No gross abnormality was found in the treated and control groups. One animal was found dead in the group fed HFD with capsaicin pellet during the dosing period, which was due to autolysis according to the necropsy analysis. Mice in the group receiving ND showed normal histology without any microscopic alterations. In all treated groups, mildly to moderately diffuse fatty infiltration was observed in the liver. No hepatitis, haemorrhage, hepatocyte necrosis and fat cysts were observed in the treated groups.

**Fig. 3:**
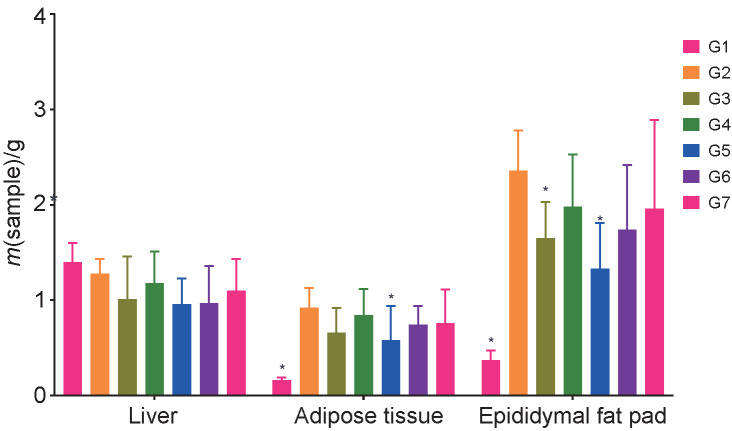
Comparison of liver, inguinal white adipose and epididymal fat pad mass. G1=normal control, G2=HFD control, G3=HFD+capsanthin-enriched pellets (low dose), G4=HFD+capsanthin-enriched pellets (medium dose), G5=HFD+capsanthin-enriched pellets (high dose), G6=HFD+capsaicin pellets, G7= HFD+orlistat, HFD=high fat diet. Values are expressed as mean±standard deviation. *Indicates statistically significant differences (p<0.05) with respect to G2, *N*=10

**Fig. 4:**
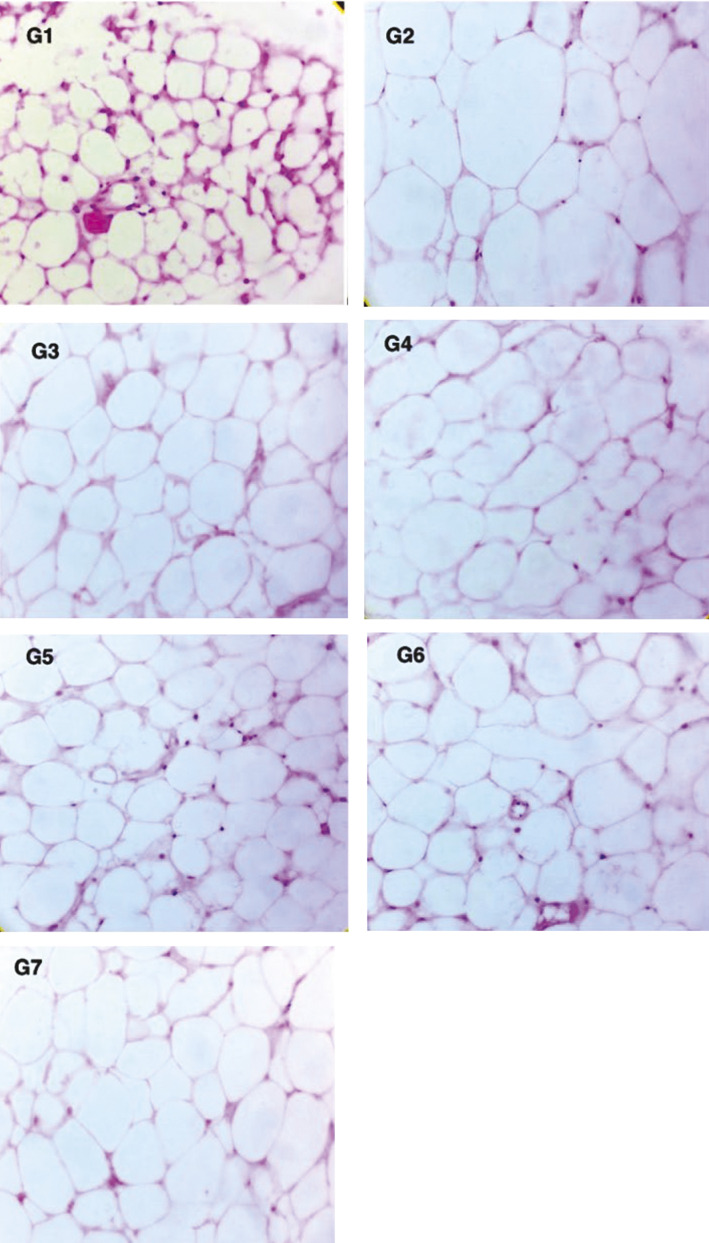
Photomicrographs of inguinal white adipose tissue (hematoxylin and eosin, magnification 40×). Smaller size of adipocytes was clearly observed in G1 (normal control). The enlarged adipocytes are observed in G2 (HFD control). Reduction in the size of adipocytes is observed in all treated groups (G3 to G7). G1=normal control, G2=HFD control, G3=HFD+capsanthin-enriched pellets (low dose), G4=HFD+capsanthin-enriched pellets (medium dose), G5=HFD+capsanthin-enriched pellets (high dose), G6=HFD+capsaicin pellets, G7=HFD+orlistat, HFD=high fat diet

**Fig. 5:**
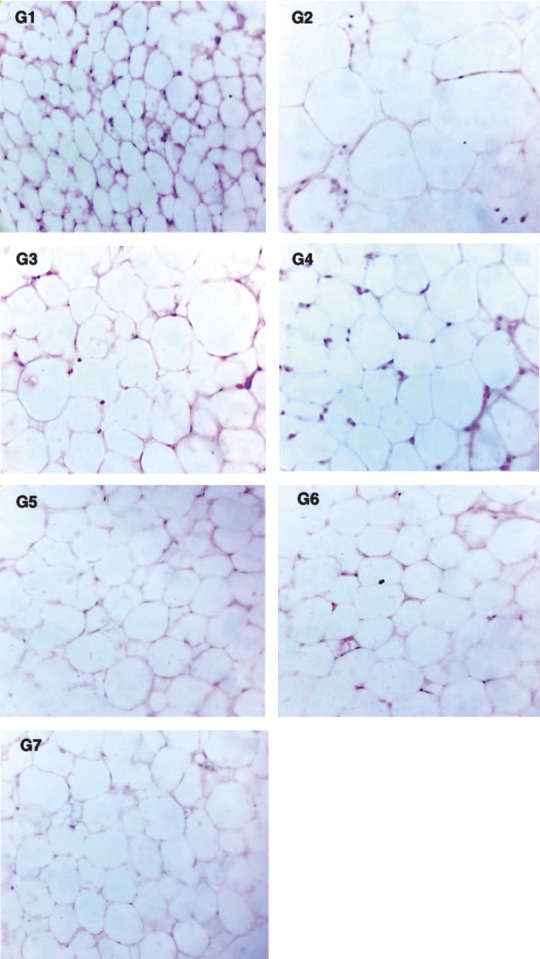
Photomicrographs of epididymal white adipose tissue (hematoxylin and eosin, magnification 40×). Smaller size of adipocytes was clearly observed in G1 (normal control). The enlarged adipocytes are observed in G2 (HFD control). Reduction in the size of adipocytes is observed in all treated groups (G3 to G7). G1=normal control, G2=HFD control, G3=HFD+capsanthin-enriched pellets (low dose), G4=HFD+capsanthin-enriched pellets (medium dose), G5=HFD+capsanthin-enriched pellets (high dose), G6=HFD+capsaicin pellets, G7= HFD+orlistat, HFD=high fat diet

The histopathological scoring of the liver is presented in Table 1. Animals treated with capsanthin-enriched pellets and capsaicin pellets did not reveal any microscopic alteration in the liver, kidneys and white adipose tissues (inguinal and epididymal). Histological examination revealed that the sizes of the white adipose tissues (inguinal and epididymal) were significantly smaller in the treated group, and a significant mass reduction in inguinal white adipose tissue (37.0% lower) and epididymal white adipose tissue (43.64% lower) was noted in the group receiving a high dose of capsanthin-enriched pellets (Fig. 3). White inguinal and epididymal adipose tissues of various treated and control groups of mice are shown in Fig. 4 and Fig. 5 respectively. The larger adipocytes were observed in the HFD group than in the ND group. In the ND group, the adipocytes were normal with regular sizes. The obese mice from the HFD control group showed fat stored in adipocytes as accumulated lipid droplets that occupy most of the cytoplasm. In the HFD group, larger adipocytes were observed in the adipose tissue than in the ND group. The normal adipocyte distribution was observed in the ND group with regular sizes of cells (Fig. 4 and Fig. 5), whereas in the HFD control, the enlarged adipocytes that occupied most of the cytoplasm were noted. However, the adipocytes of the groups receiving HFD with capsanthin-enriched pellets and HFD with capsaicin pellets were smaller and comparable to the groups fed HFD with orlistat and ND.

**Table 1:** Histopathological scoring of liver

Observation^a^	G1	G2	G3	G4	G5	G6	G7
Fatty infiltration	-	+++	+	+	++	++	++
Hypertrophy of adipocytes/inguinal white adipose tissue	-	+++	+	+	++	++	++
Hypertrophy of adipocytes/epididymal white adipose tissue	-	+++	+	+	++	++	++
Hepatitis	-	++	-	-	-	-	-
Haemorrhage	-	-	-	-	-	-	-
Hepatocyte necrosis	-	-	-	-	-	-	-
Fat cysts	-	-	-	-	-	-	-

^a^The severity was evaluated based on the following scoring scheme: -=normal, +=mild effect, ++=moderate effect, +++=severe effect. Values are expressed as mean±standard deviation (*N*=10). G1=normal control, G2=HFD control, G3=HFD+capsanthin-enriched pellets (low dose), G4=HFD+capsanthin-enriched pellets (medium dose), G5=HFD+capsanthin-enriched pellets (high dose), G6=HFD+capsaicin pellets, G7=HFD+orlistat, HFD=high fat diet

### Haematology and clinical chemistry

Blood acts as a pathological reflector of the status of the animals exposed to phytochemicals and plays a vital role in their biological, pathological and nutritional status (*46*). The examination of haematological parameters provides the opportunity to clinically investigate the impact of highly standardized capsanthin, which has not been assessed and reported previously. We examined the clinical chemistry and haematological parameters and reported the obesity biomarkers of mice fed capsanthin-enriched pellets for the first time. There was no significant change in the haematological (Table S5) and clinical chemistry parameters (Table 2) in all control and treated groups. However, a nonsignificant reduction of the total cholesterol, LDL, HDL, triglycerides and blood glucose levels was observed in the groups treated with capsanthin-enriched pellets compared to the control group receiving HFD.

**Table 2:** Clinical chemistry parameters

*γ*(parameter)/(mg/100 mL)	G1	G2		G3		G4		G5			G6		G7	
GLU	180±220		217±24		169±23		206±62		206±40	198±25		215±69	
CHO	(70.9±7.8)^a^		124.6±9.9		114±27		131±40		115±18	118±20		120±25	
TRI	48±15		64±22		70±17		81±25		94±39	85±22		76±25	
HDL	(38.5±5.4)^a^		63.3±3.4		56.0±12.6		62.8±19.4		59.0±11.0	60.5±10.6		54.6±18.1	
LDL	3.0±0.4		4.5±0.7		5.5±3.1		4.4±1.9		4.7±0.7	4.6±1.1		6.6±4.9	
URE	43.2±6.3		48.3±7.8		50.6±5.5		52.8±3.4		50.4±5.6	55.1±7.4		53.3±5.2	
CRE	0.38±0.03		0.38±0.02		0.36±0.02		0.37±0.02		0.39±0.02	0.39±0.02		0.40±0.01	
*N*(parameter)/(U/L)													
AST	71±22		132±72		88±37		176±89		92±40	110±51		126±62	
ALT	33.6±1.6		44±12		31±13		48±11		33±5	33.2±6.2		36±14	

Values are expressed as mean±standard deviation (*N*=10). ^a^Indicates statistically significant differences (p<0.05) with respect to G2. G1=normal control, G2=HFD control, G3=HFD+capsanthin-enriched pellets (low dose), G4=HFD+capsanthin-enriched pellets (medium dose), G5=HFD+capsanthin-enriched pellets (high dose), G6=HFD+capsaicin pellets, G7=HFD+orlistat, HFD=high fat diet, S.D.=standard deviation, Glu=glucose, CHO=cholesterol, TRIG=triglycerides, HDL=high density lipoprotein, LDL=low density lipoprotein, URE=urea, CRT=creatinine, AST=aspartate aminotransferase, ALT=alanine aminotransferase, TP=total protein, ALB=albumin, ALP=alkaline phosphatase

## CONCLUSIONS

In this study, we demonstrated that highly enriched and stabilized capsanthin-enriched pellets given to all groups of mice in low, medium and high doses significantly reduced mass gain and that these effects are comparable to capsaicin pellets and the reference drug orlistat. The serum, glucose, total cholesterol, LDL and HDL triglyceride contents were lower in the groups treated with capsanthin-enriched pellets and capsaicin pellets than in the control group fed high-fat diet (HFD), whereas the obesity marker adiponectin was significantly higher in the groups fed capsanthin-enriched pellets and capsaicin pellets. The groups receiving capsanthin-enriched pellets and capsaicin pellets had a significantly reduced concentrations of leptin, free fatty acids and insulin. There was no change in the liver mass in all groups, but there was a significant reduction in adipose tissues (inguinal and epididymal white) in all the mice receiving capsanthin-enriched pellets and capsaicin pellets compared to the HFD group. The anti-obesity effect of capsanthin-enriched pellets is remarkably significant compared to the previous findings. The reason may be due to the highest concentration of capsanthin in our capsanthin-enriched pellets. There was no change in haematological and biochemical parameters across the study groups. Therefore, the oral administration of capsanthin-enriched pellets and capsaicin pellets significantly reduces body mass gain, increases adiponectin, and decreases leptin, free fatty acid and insulin concentrations without any adverse effects at the tested doses. These results suggest that capsanthin-enriched extract can be used as an alternative natural phytochemical in the development of functional foods with potential anti-obesity activities. Further gene expression studies should be conducted to better understand the mechanism of the highly enriched capsanthin.

## Figures and Tables

**Table S1:** Composition of capsanthin-enriched pellets

Matrix	Ingredient	*m*(ingredient)/g
Core	capsanthin-enriched extract	1000
	pharmaceutical-grade sucrose	200
	microcrystalline cellulose	200
	polyvinyl pyrrolidine K 30 (5%) in isopropyl alcohol	SQ
Coating	ethyl cellulose N-20	20
	sodium alginate	20
	ethanol	SQ
	water	SQ

SQ=sufficient quantity

**Table S2:** Specification of capsanthin-enriched pellets

Parameter	Specification	Method
Physical description	dark red pellets	Organoleptic
Identification	identified by UV	(*22*)
Solubility	slightly soluble in alcohol, insoluble in water	(*19*)
*d*(particle)/mesh	30 (90 % passes through)	(*20*)
*w*(loss on drying)/%	<2.0	(*21*)
Chemical assay		
*w*(capsanthin)/%	>50.0	(*22*)
*w*(carotenoids)/%	>60.0	(*22*)
*w*(impurity)/(mg/kg)		
Lead	<5.0	(*23*)
Arsenic	<3.0	(*23*)
Cadmium	<1.0	(*23*)
Mercury	<1.0	(*23*)
*N*(microorganism)/(CFU/g)		
Total aerobic microbial count	<3000	(*24*)
Total yeast and mould count	<1.0	(*24*)
*Escherichia coli *	negative in 10 g of sample	(*25*)
*Salmonella* species	negative in 10 g of sample	(*25*)
*Staphylococcus aureus*	negative in 10 g of sample	(*25*)
*Pseudomonas aeruginosa*	negative in 10 g of sample	(*26*)

UV=ultraviolet spectrophotometer, CFU=colony forming unit

**Table S3:** Room temperature stability data for capsanthin-enriched extract

Parameter	Specification	*t*/month			
0	3	6	12
Description	Dark red pellets	Dark red pellets	Same as initial	Same as initial	Same as initial
Identification	Identification by UV	Identified	Identified	Identified	Identified
*w*(loss on drying)/%	1.24	1.32	1.88	1.52	1.79
*w*(capsanthin)/%	54.21	54.19	54.09	53.88	53.97
*w*(carotenoids)/%	62.86	62.99	61.79	62.16	62.58
Change of colour	-	No	No	No	No

Storage conditions: temperature=(25±2) °C, relative humidity=(60±5) %. UV=ultraviolet spectrophotometer

**Table S4:** Summary of feed consumption

*t*/day	Food consumption/(g/day)						
G1	G2	G3	G4	G5	G6	G7
1–3	(4.2±0.5)^a^	2.7±0.3	2.4±0.4	2.5±0.3	2.5±0.5	2.5±0.7	2.7±0.3
3–6	(4.0±0.5)^a^	2.8±0.2	2.6±0.3	2.7±0.3	2.5±0.6	2.3±0.5	2.8±0.3
6–8	(4.0±0.4)^a^	3.0±0.4	2.8±0.7	(2.2±0.8)^a^	(2.3±0.7)^a^	2.9±0.7	2.6±0.4
8 10	(4.0±0.4)^a^	2.9±0.5	2.7±0.4	2.5±0.5	2.7±0.3	3.0±0.5	2.8±0.5
10–13	(3.8±0.2)^a^	2.9±0.3	2.8±0.2	2.7±0.5	(2.5±0.4)^a^	2.9±0.4	2.6±0.4
13–15	(3.7±0.6)^a^	3.0±0.3	2.7±0.3	2.6±0.5	2.4±0.6	3.1±0.6	2.7±0.6
15–17	(3.7±0.3)^a^	2.9±0.3	2.6±0.3	2.7±0.4	2.5±0.4	2.7±0.6	2.9±0.4
17–20	(4.1±0.5)^a^	2.9±0.5	2.7±0.2	2.9±0.5	2.7±0.2	2.7±0.6	2.8±0.5
20–22	(4.9±0.3)^a^	3.0±1.1	2.9±0.8	3.3±1.4	2.7±0.8	2.9±0.9	2.8±0.8
22–24	(4.1±0.5)^a^	2.8±0.5	3.0±0.4	2.9±0.3	2.9±0.4	3.1±0.3	3.0±0.3
24–27	(4.3±0.5)^a^	2.9±0.4	2.9±0.3	3.0±0.3	2.8±0.2	3.0±0.2	3.1±0.3
27–29	(4.9±0.3)^a^	3.0±0.3	3.0±0.3	3.0±0.4	2.9±0.4	3.0±0.2	3.1±0.2
29–31	(4.3±0.5)^a^	3.0±0.2	2.8±0.4	3.0±0.7	2.8±0.4	3.0±0.3	3.1±0.2
31–34	(4.1±0.3)^a^	3.0±0.3	2.9±0.3	2.9±0.3	3.0±0.3	3.3±0.7	3.1±0.3
34–36	(4.2±0.4)^a^	3.1±0.4	3.1±0.2	3.2±0.3	3.0±0.3	3.1±0.3	3.2±0.2

Values are expressed as mean±standard deviation (*N*=10). ^a^Indicate statistically significant differences (p<0.05) with respect to G2. G1=normal control, G2=HFD control, G3=HFD+capsanthin-enriched pellets (low dose), G4=HFD+capsanthin-enriched pellets (medium dose), G5=HFD+capsanthin-enriched pellets (high dose), G6=HFD+capsaicin pellets, G7=HFD+orlistat, HFD=high fat diet

**Table S5:** Haematological parameters

Parameter		G1	G2	G3	G4	G5	G6	G7
*N*(WBC)/(10^3^/μL)		5.8±1.1	3.8±1.1	4.6±1.1	4.7±1.5	4.6±0.8	3.7±0.8	4.6±0.4
*N*(RBC)/(10^6^/μL)		10.7±1.5	12.7±0.7	12.6±1.5	12.2±0.5	11.4±1.7	(10.7±0.8)^a^	10.8±1.0
*γ*(Hb)/(g/100 mL)		(14.4±1.9)^a^	17.3±0.6	16.1±2.1	16.0±0.5	15.4±2.2	(14.6±1.0)^a^	(14.5±1.5)^a^
*w(*(HCT)/%		52.3±7.3	61.4±3.2	61.1±7.3	58.8±2.3	54.72±8.18	51.56±4.03	51.74±5.23
*N*(PLT)/(10^3^/μL)	1377±286		768±145	1165±234	1224±266	1501±114	1464±188	1551±15
*V*(MC)/µm^3^		49.0±0.0	48.4±0.6	48.2±0.5	48.4±0.6	47.8±0.5	48.2±0.5	48.2±0.8

*m*(MCH)/pg		13.4±0.2	13.7±0.3	(12.7±0.5)^a^	13.2±0.2	13.7±0.2	13.7±0.2	13.9±0.9	
*γ*(MCHC)/(g/dL)		27.7±0.4	28.2±0.5	(26.3±0.9)^a^	(27.2±0.4)^a^	28.1±0.3	28.4±0.4	28.0±0.3	
*w*(NEU)/%		23.6±2.2	26.0±4.0	27.6±3.6	24.0±2.8	26.0±2.8	28.8±3.6	25.6±3.3	
*w*(LYM)/%		70.4±2.6	69.6±3.3	69.6±1.7	72.0±2.0	70.0±1.4	67.6±2.2	69.2±1.1	
*w*(EOS)/%		2.8±0.9	2.2±0.8	1.2±1.1	1.4±1.1	3.0±1.9	1.8±0.8	2.2±1.3	
*w*(MON)/%		3.2±0.8	2.2±0.8	1.6±1.3	2.6±1.1	3.0±1.9	1.8±1.3	3.0±1.9	
*w*(BAS)/%		0.00±0.00	0.00±0.00	0.00±0.00	0.00±0.00	0.00±0.00	0.00±0.00	0.00±0.00	

Values are expressed as mean±standard deviation (*N*=10). ^a^Indicates statistically significant differences (p<0.05) with respect to G2. G1=normal control, G2=HFD control, G3=HFD+capsanthin-enriched pellets (low dose), G4=HFD+capsanthin-enriched pellets (medium dose), G5=HFD+capsanthin-enriched pellets (high dose), G6=HFD+capsaicin pellets, G7=HFD+orlistat, HFD=high fat diet, WBC=total leukocyte count, RBC=erythrocyte count, Hb=haemoglobin, HCT=haematocrite, PLT=platelet, MC=mean corpuscular, MCH=mean corpuscular haemoglobin, MCHC=mean corpuscular haemoglobin concentration, NEU=neutrophils, LYM=lymphocytes, EOS=eosinophils, MON=monocytes, BAS=basophils
